# Modulation of individual auditory-motor coordination dynamics through interpersonal visual coupling

**DOI:** 10.1038/s41598-017-16151-5

**Published:** 2017-11-24

**Authors:** Kohei Miyata, Manuel Varlet, Akito Miura, Kazutoshi Kudo, Peter E. Keller

**Affiliations:** 1 Department of Life Sciences, Graduate School of Arts and Sciences, The University of Tokyo, Tokyo, Japan; 2 Japan Society for the Promotion of Science, Tokyo, Japan; 3 The MARCS Institute for Brain, Behaviour and Development, Western Sydney University, NSW, Australia; 4 Faculty of Sport Sciences, Waseda University, Saitama, Japan

## Abstract

The current study investigated whether visual coupling between two people producing dance-related movements (requiring whole-body auditory-motor coordination) results in interpersonal entrainment and modulates individual auditory-motor coordination dynamics. Paired participants performed two kinds of coordination tasks – either knee flexion or extension repeatedly with metronome beats (Flexion-on-the-beat and Extension-on-the-beat conditions) while standing face-to-face or back-to-back to manipulate visual interaction. The results indicated that the relative phases between paired participants’ movements were closer to 0° and less variable when participants could see each other. In addition, visibility of the partner reduced individual differences in the dynamics of auditory-motor coordination by modulating coordination variability and the frequency of phase transitions from Extension-on-the-beat to Flexion-on-the-beat. Together, these results indicate that visual coupling takes place when paired participants can see each other and leads to interpersonal entrainment during rhythmic auditory-motor coordination, which compensates for individual differences via behavioural assimilation and thus enables individuals to achieve unified and cohesive performances.

## Introduction

Group dancing and music ensemble performance are widespread social behaviours that involve temporally precise interpersonal synchronisation based on information exchanged via multiple sensory modalities. Collective action during these group activities enables performers to create complex and enjoyable works that transcend what can be achieved via solo performance. Moreover, such coordinated behaviour can have potent pro-social effects, as evidenced by a growing body of research showing that synchronising movements with others promotes interpersonal affiliation and cooperation^1–4^. Culturally and socially significant behaviours are thus founded on a basic human capacity for interpersonal synchronisation, that is, the coupling of rhythmic movements between individuals through auditory, visual, and tactile channels^5,6^. In the context of group dance and music, the auditory modality is often considered to be paramount, as it is well suited to providing a salient rhythmic beat that can be used as a common temporal reference to support interpersonal synchronisation of body movements in large groups and under conditions where visual coupling is not continuously available. Research on interpersonal coupling via the visual modality has, however, shown that people have a spontaneous tendency to synchronise with each other’s movements when they can see one another, referred to as interpersonal entrainment, and that this tendency can be difficult to resist^7^. In the current study, we investigated the effects of interpersonal visual coupling on auditory-motor coordination in a dance-related task. Specifically, we examined whether the vision of a partner affects the individual coordination of simple dance movements with auditory rhythms.

Numerous studies have demonstrated that paired participants producing rhythmic movements tend to spontaneously synchronise their movements when they can see each other. This has been shown in contexts ranging from simple experimental tasks, such as finger oscillation^8^ and swinging hand-held pendulums^9^, to more ecological conditions, such as gait^10,11^, postural coordination^12^ and rocking-chair swaying^13,14^. Specifically, the relative phase between individuals’ movements is typically closer to 0° (in-phase) or 180° (antiphase), with in-phase attraction being the strongest. However, Shockley and colleagues^15^ reported no effect of visual interaction on interpersonal postural entrainment when two participants conversed freely standing face-to-face while playing a spot-the-difference puzzle game. Previous studies have suggested high rhythmicity and large amplitude of movements facilitate spontaneous visuo-motor coordination^16–18^. Free conversation does not have apparent periodic rhythm, but the majority of dance and music performances do. Together, the above findings suggest that visual coupling can cause interpersonal entrainment in group musical activities. Furthermore, by modulating the phase and frequency of individual rhythmic movements, such visual coupling and interpersonal entrainment with a partner, could affect each individual’s ability to coordinate with an external auditory or musical rhythm.

Movement coordination with external auditory rhythms plays a crucial role in dance and music performance^19–22^. Such auditory-motor coordination has often been investigated through dynamical systems theory and methods. Early application of this approach to human motor control is found in the seminal work of Kelso and colleagues on the dynamics of rhythmic bimanual coordination^23,24^. Studies using this approach to auditory-motor coordination have reported that coordination stability changed depending on the movement frequency and phase relation between the beats of a metronome and the movement produced^25,26^. Metronome beats and movements were stably coordinated for both in-phase and antiphase at slow frequencies. Systematic increases in beat frequency induced abrupt changes from antiphase to in-phase coordination, referred to as phase transitions. Musical experience modulated these effects, with expert musicians and dancers producing more stable auditory-motor coordination than non-experts^19–22,25,27,28^. In the studies of Miura *et al*.^19,25^, dancers and non-dancers were instructed to perform two patterns of coordination with metronome beats while producing rhythmic whole-body movements – flexion-on-the-beat and extension-on-the-beat. These patterns represent basic forms of movement coordination with music used in street dancing. In the flexion-on-the-beat condition, participants were required to flex their knees with each beat of the metronome while keeping a standing posture. In the extension-on-the-beat condition, participants were required to extend their knees with each beat of the metronome. The results of these studies showed that, although phase transitions from the extension-on-the-beat pattern to flexion-on-the-beat pattern occurred as beat frequency increased for both groups, dancers could maintain the extension-on-the-beat pattern at higher movement frequencies than non-dancers. In addition, both coordination patterns were less variable in dancers compared with non-dancers. These findings suggest that rhythmic ability and expertise are reflected in the dynamics of auditory-motor coordination.

However, previous research has not investigated whether such individual auditory-motor coordination—despite being often produced in social contexts—is modulated by visual coupling and the occurrence of interpersonal entrainment. Specifically, it has not been tested whether the efficiency of auditory-motor coordination is influenced by the vision of co-performers. Indirect support for this possibility is provided by a study by Varlet *et al*.^12^ that examined postural sway in the sagittal plane of paired participants who could see or could not see each other. This study did not investigate individual auditory-motor coordination but examined postural coordination between the ankle and the hip. In postural coordination, phase transitions typically occur from in-phase (ankle and hip joints oscillating in the same direction) to antiphase (ankle and hip joints oscillating in opposite directions) when movement frequency increased^29^. Particularly interesting in the present context is that the results of Varlet *et al*.^12^ demonstrated that the frequencies at which phase transition occur became more similar when there was visual interaction, indicating that participants tended to match their postural coordination. By extension, the dynamics of auditory-motor coordination, and more specifically transition frequencies from extension-on-the-beat to flexion-on-the-beat, could be modulated by visual coupling and the occurrence of interpersonal entrainment, because interpersonal entrainment encourages paired participants to match their auditory-motor coordination pattern.

Varlet *et al*.^12^ also demonstrated that visual interaction increases the variability of individual visual and postural coordination. To control the frequency of individuals’ postural sway, participants were asked to lean forward and backward in order to keep a constant distance between their head and a visual target that oscillated in the sagittal plane. The variability of coordination between each participant’s postural sway and the visual target increased when participants could see each other, despite the fact that the targets of the two participants oscillated in perfect synchrony. This finding suggests visual coupling increases the variability of individual sensorimotor coordination.

A possible reason for this increased variability is that spontaneous interpersonal coupling makes individuals susceptible to a partner’s movement fluctuations or variability. The variability of auditory-motor coordination could therefore increase when performed with a visible partner. On the other hand, it is possible that the individual-beat coupling and individual-individual coupling could reinforce each other, and that the variability of auditory-motor coordination could therefore decrease. This possibility is supported by previous studies showing that coupled action systems acting as a single functional unit or coordinative structure can enhance temporal stability^30,31^. A related study on dance also reported that participants’ sensorimotor coordination with music was improved when they could see each other^32^.

In addition, the effects of interpersonal entrainment on auditory-motor coordination could differ depending on individual coordination skills. Strong auditory-motor coupling, which is characterized by less variable coordination and high adaptability (error correction), is a hallmark of coordination skill^19,22,33^, and such skill could thus make individuals less susceptible to the effects of interpersonal entrainment. Therefore, it is necessary to evaluate the effect of visual coupling depending on individual coordination skill.

Based on previous research, it is therefore known that visual coupling between individuals can lead to interpersonal entrainment, and that this can affect intra-personal coordination dynamics. However, it is still unknown whether these effects extend to situations where auditory-motor coordination is simultaneously required, which is crucial in music and dance performance. The goal of the present study is hence to investigate whether visual coupling between paired individuals engaged in a dance-related task (1) results in the occurrence of interpersonal entrainment and (2) modulates individual dynamics of auditory-motor coordination. To balance ecological validity and experimental control, we employed a task that isolated a single component of a complex motor skill typically performed with others: a knee flexion and extension motion from the street dancing movement repertoire^19,25^. In the present study, we calculated relative phase angle between an individual’s periodic movement and another individual’s movement or metronome beats because it has often been used to assess temporal precision in coordination^10,13,25^. The variability of phase angles, in particular, is typically considered to be an inverse measure of coordination stability^19^. At the interpersonal level, we hypothesized that the relative phase between the movements of two visually coupled participants would be closer to 0° and less variable, indicating the occurrence of interpersonal entrainment between individuals. At the individual level, it is expected that visually coupled participants tend to match their phase transition points (from extension-on-the-beat to flexion-on-the-beat) and their variabilities of auditory-motor coordination, resulting in positive or negative effects on auditory-motor coordination depending on individual coordination skills.

## Methods

### Participants

Thirty-two healthy volunteers (25 females and 7 males; mean age 21.97 years, range 18–43 years) from the Western Sydney University community participated in this study. Their mean height was 165.72 cm (*SD* = 8.23) and average weight was 65.13 kg (*SD* = 15.61). They were assigned to 7 mixed pairs and 9 female pairs randomly or with their friends on a case-by-case basis. Additional analyses conducted on gender-match and familiarity between participants are reported in Supplemental Information (see Supplementary Tables S1–S3). The mean of within-pair height difference was 10.06 cm (*SD* = 6.81) and the mean of within-pair mass difference was 18.13 kg (*SD* = 13.38). This study conformed to the Declaration of Helsinki, and informed consent was obtained from all participants. This study was approved by the Western Sydney University Human Research Ethics Committee.

### Task and Procedures

Paired participants were instructed to perform knee flexion and extension to the beat while keeping a standing posture without moving any other parts of their body apart from the hip, knee, and ankle joints. They performed the task in two different coordination conditions (Flexion-on-the-beat and Extension-on-the-beat) and two different orientation conditions (Face-to-face and Back-to-back). Both participants in a pair received the same flexion and extension instruction on each trial. They were not explicitly instructed to coordinate with each other. In all conditions, participants were asked to look forward and to do their best to coordinate their movement with the auditory metronome beats.

The distance between paired participants was 200 cm. Metronome beats (trains of 80 ms sine wave pulses, carrier frequency 440 Hz) were presented via four speakers. The frequency was increased from 80 to 160 beat per minutes (bpm) in steps 10 bpm, every 16 beats. Four additional beats served as a “ready” cue at the beginning of each trial. Although auditory stimuli were discrete beats, participants were asked to keep moving continuously as when dancing. The task duration in each trial was 75 seconds. Participants performed 20 trials (2 auditory-motor coordination conditions × 2 orientation conditions × 5 repetitions). The order of trials was randomized.

Before the experimental recording, participants were fitted with motion capture markers (see below) and practiced the tasks for a few minutes. Participants were instructed to keep a one-to-one correspondence between their movement and the beat, and not to resist any change in coordination pattern. They were asked to always perform the most comfortable pattern of coordination.

### Data acquisition and Analysis

**Figure 1:**
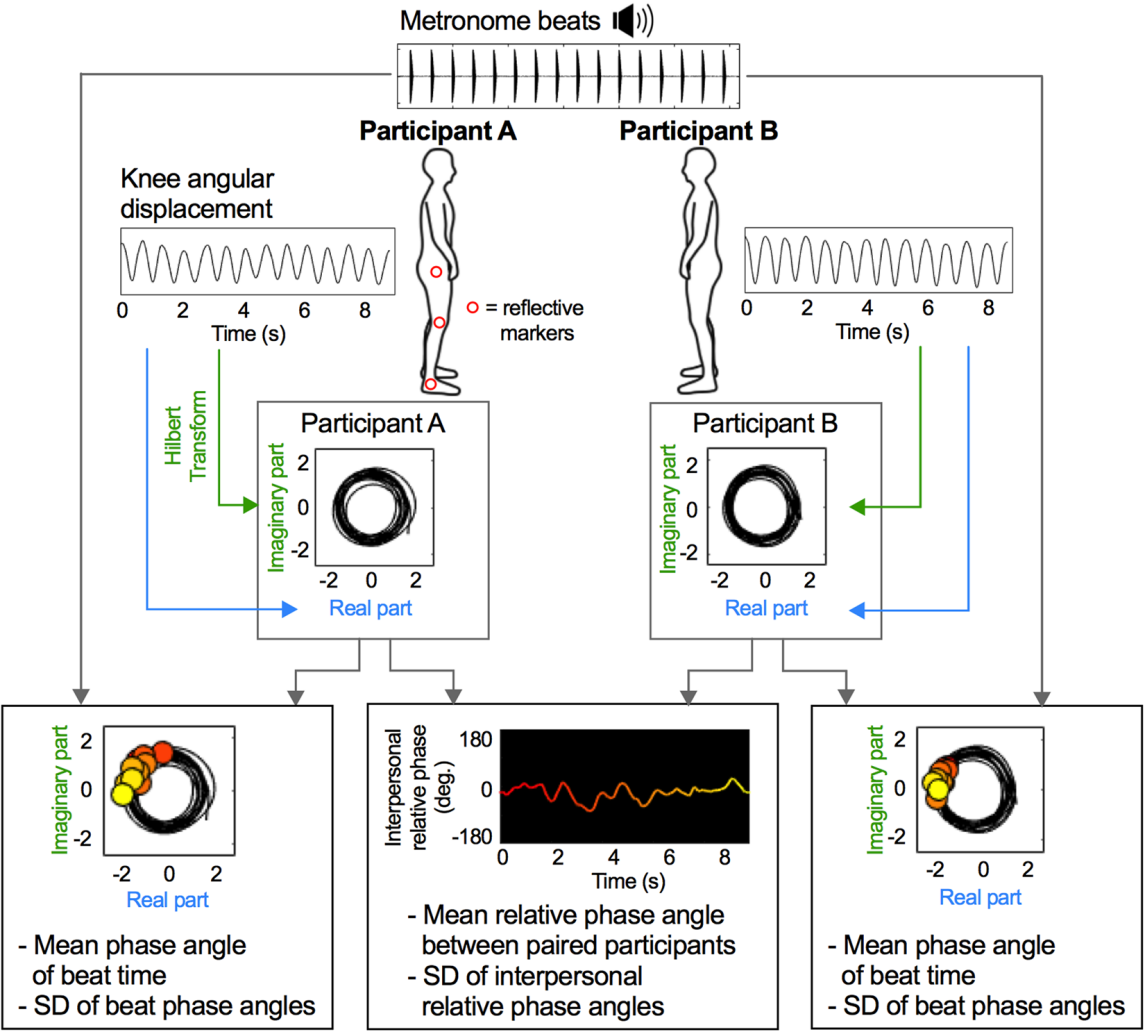
Example of data processing flow at 80 bpm in the Flexion-on-the-beat and Face-to-face condition. To record knee angle displacement, three reflective markers were attached to the right centre of hip, knee, and ankle joints (and are therefore visible on Participant A but not Participant B). Time series data of knee angle displacement were low-pass filtered (cut-off frequency = 7 Hz). The continuous phase of each participant’s movement was computed using the Hilbert Transform. Both the real and imaginary parts of displacement obtained by the Hilbert Transform were normalized and plotted on a phase plane (with the real part of displacement on the X-axis and the imaginary part of displacement on the Y-axis) to compute the continuous phase of each participant’s movement. The mean and standard deviation (SD) of interpersonal phase angles were then calculated within each trial using circular statistics. Audio signals of the metronome recorded in synchrony with the motion capture data were analysed to determine the beat onset times. Individual beat onset times were then superposed on each phase plane trajectory to compute the mean phase angle of beat time and its SD using circular statistics. We defined maximum flexion as 0° and maximum extension as 180° because the most stable coordination pattern is conventionally defined as 0° or in-phase, and previous studies have reported that flexion-on-the-beat pattern is the most stable pattern. Colour of interpersonal phase angle and beat markers changes from red to yellow with time-course.

The data processing flow is shown in Fig. 1. Participants’ movements were measured using a Vicon 12-camera motion capture system (Vicon Motion Systems Ltd., Oxford, UK). Knee angular displacement, assumed to be representative of whole-body vertical movement, was calculated from markers positioned on the right hip, knee and ankle joint centres of participants. Displacement data were low-pass filtered with a bidirectional second-order Butterworth filter (cut-off frequency = 7 Hz). The first three movement cycles of each frequency plateau were discarded to remove the transient effects due to frequency change. The continuous phase of each participant’s movement was computed using the Hilbert Transform^34^. Both the real and imaginary parts of displacement obtained by the Hilbert Transform were normalized by calculating Z-values. The mean and standard deviation (SD) of the relative phase angles between the two participants were calculated within each trial using circular statistics^35^. The within-trial mean relative phase was converted to an absolute value because leader and follower relations were not relevant to the aims of this study. We then calculated mean values across pairs of the mean and SD of relative phase angles between paired participants. In addition, to confirm that changes in interpersonal phase relation (mean and SD) were due to the visual coupling in the Face-to-Face condition and not other factors such as changes in attention load or internal motor representation of the partner, for instance, we conducted a control analysis with permutations of the trials. In this analysis, the interpersonal phase angles were calculated between the data of each participant’s movement and the data of their partner in a different trial from the Face-to-face condition for the corresponding coordination pattern (Face-pseudo). The mean and SD of relative phase angles between paired participants were then calculated for all possible combinations within a pair apart from the actual trial combination, and compared with those calculated from the actual trial combination in the Face-to-face condition (Face-real) and in the Back-to-back condition (Back-real). Finding that interpersonal phase relations differ between Face-real and Face-pseudo data, but not between Back-real and Face-pseudo data, would indicate that any differences between the Face-to-face and Back-to-back conditions in the main analyses are attributable to visual coupling.

Audio signals of the metronome recorded in synchrony with the motion capture data were analysed to determine the beat onset times. Individual beat onset times were then superposed on each phase plane trajectory to compute the mean phase angle of beat time and its SD using circular statistics. We defined maximum flexion as 0° and maximum extension as 180°. The range from −180° to 0° is the flexion phase, and the range from 0° to 180° is the extension phase. The mean and SD of beat phase angles were then each averaged across trials.

To test the hypothesis that the effect of visual coupling could differ depending on individual coordination skill, we divided individual participants into a good coordination group (less variable) and a poor coordination group (more variable) based on the SD of beat phase angles in the Back-to-back condition using a median split. The mean and SD of beat phase angles, which were calculated across trials, were then averaged across participants separately for each group. The correlation was calculated between the SD of beat phase angles in the Back-to-back condition and the changes in SD of beat phase angles, which were determined by subtracting the variability in the Back-to-back condition from the variability in the Face-to-face condition. Negative values indicate a decrease in variability with visual coupling whereas positive values indicate an increase in variability with visual coupling.

The frequency at which the phase transition occurred (phase transition frequency) was defined as the first moment at which the coordination pattern deviated from the Extension-on-the-beat pattern. The deviation was determined when at least 3 successive phase angles of beat time fell into the Flexion-on-the-beat range. We calculated the average phase angle of the Flexion-on-the-beat pattern across all participants and beat rates in the Back-to-back condition, and then defined the range of $\pm$90° from the average as the Flexion-on-the-beat range. The average angle was about −47° (−47.21°), so we set the flexion-on-the-beat range from −137° to 43°. When there was no phase transition within the tested beat rate, the transition frequency was defined as 170 bpm, because previous research has demonstrated that highly skilled dancers show transitions at around 170 bpm on average^25^. The absolute difference between transition frequencies within a pair was calculated to investigate whether paired participants match their dynamics of auditory-motor coordination. To explore the effect of visual coupling depending on participants’ inherent transition frequencies, the correlation was calculated between transition frequency in the Back-to-back condition and the changes in transition frequency due to visual coupling, which were defined by subtracting the transition frequency in the Back-to-back condition from the frequency in the Face-to-face condition.

We excluded 16 of 320 trials (5%) from analyses because we could not identify markers in offline labelling for two trials and participants failed to maintain a 1:1 frequency relation for 14 trials. The datasets in the current study are available from the corresponding author upon request.

### Statistics

Separate 3-way analyses of variance (ANOVAs) with three within-subject factors, coordination pattern (Flexion-on-the-beat and Extension-on-the-beat), orientation (Face-to-face and Back-to-back), and beat rate (from 80 to 160 bpm in steps of 10 bpm) were performed on (1) the mean relative phase and (2) the mean SD of relative phase between the two participants. In addition, separate 3-way ANOVAs with three within-subject factors, permutation (Face-real, Back-real, and Face-pseudo), coordination pattern, and beat rate were performed on (1) the mean relative phase and (2) the mean SD of relative phase between the two participants. Separate 4-way ANOVAs with one between-subject factor, group (good coordination group and poor coordination group), and three within-subject factors, coordination pattern, orientation, and beat rate were performed on (1) the mean phase angle of beat time, and (2) the mean SD of beat phase angles. The Greenhouse-Geisser correction was used in cases where Mauchly’s test of sphericity was significant. Tests of simple effects were performed to follow up significant interactions. A Bonferroni correction was used to correct for multiple comparisons. A paired t-test was employed to compare the absolute difference between transition frequencies within a pair across orientation conditions. Separate Pearson’s correlation coefficients were computed to assess the relationship (1) between inherent variability of auditory-motor coordination and changes in variability with visual coupling, (2) between inherent transition frequency and changes in transition frequency with visual coupling. For all analyses, the statistical significance level was set at *p* < 0.05.

## Results

### Interpersonal phase relations

**Figure 2:**
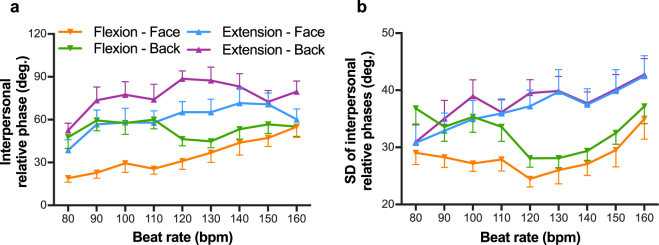
Mean relative phase angle between paired participants (**a**) and mean standard deviation of interpersonal relative phase angles (**b**) as a function of beat rate, which increased from 80 to 160 bpm. Vertical bars represent between-participant standard error. Lower error bars are for the Flexion-on-the-beat condition, and upper error bars are for the Extension-on-the-beat condition.

Figure 2a shows the mean relative phase between paired participants as a function of beat rate. The ANOVA on these data showed a significant main effect of coordination pattern [*F*(1, 15) = 63.42, *p* < 0.001, ${\eta }_{p}^{2}$ = 0.81], orientation [*F*(1, 15) = 38.94, *p* < 0.001, ${\eta }_{p}^{2}$ = 0.72], and beat rate [*F*(3.12, 46.86) = 3.36, *p* = 0.03, ${\eta }_{p}^{2}$ = 0.18], and a significant three-way interaction [*F*(8, 120) = 2.36, *p* = 0.02, ${\eta }_{p}^{2}$ = 0.14]. We divided the 3-way ANOVA into 2-way analyses of orientation × coordination pattern at each level of beat rate, coordination pattern $\times$ beat rate at each level of orientation condition, and orientation $\times$ beat rate at each level of coordination pattern. The interaction of orientation × coordination pattern was significant at 90 bpm [*F*(1, 15) = 5.14, *p* = 0.04]. Tests of simple effects showed that, for the Face-to-face condition, the relative phase was significantly closer to 0° in the Flexion-on-the-beat condition than in the Extension-on-the-beat condition [*F*(1, 15) = 11.05, *p* < 0.01], but not for the Back-to-back condition. There were no significant two-way interactions between coordination pattern and beat rate at each level of orientation condition. The phase relations between paired participants were significantly closer to 0° in the Flexion-on-the-beat condition than in the Extension-on-the-beat condition in both the Face-to-face condition [*F*(1, 15) = 36.17, *p* < 0.001] and the Back-to-back condition [*F*(1, 15) = 33.84, *p* < 0.001]. There were significant two-way interactions between orientation and beat rate in the Flexion-on-the-beat condition [*F*(8, 120) = 5.78, *p* < 0.001] but not in the Extension-on-the-beat condition. Simple effect tests revealed that, for Flexion-on-the-beat, the relative phase in the Face-to-face condition was significantly closer to 0° than in the Back-to-back condition for beat rates from 80 to 120 bpm (*ps* < 0.01). These results indicate that, as predicted, the phase relations between paired participants were closer to 0° while visual interaction, especially at lower beat rates.

Figure 2b shows the mean SD of interpersonal phase relation as a function of beat rate. The main effect of coordination pattern and orientation condition was significant [*F*(1, 15) = 31.63, *p* < 0.001, ${\eta }_{p}^{2}$ = 0.68 and *F*(1, 15) = 17.83, *p* < 0.01, ${\eta }_{p}^{2}$ = 0.54, respectively], but the effect of beat rate and three-way interaction were not significant [*F*(2.84, 42.67) = 2.49, *p* = 0.08 and *F*(8, 120) = 0.41, *p* = 0.91, respectively]. There were significant two-way interactions between coordination pattern and beat rate [*F*(3.27, 49.03) = 4.87, *p* < 0.01, ${\eta }_{p}^{2}$ = 0.25] and between orientation and coordination pattern [*F*(1, 15) = 5.00, *p* = 0.04, ${\eta }_{p}^{2}$ = 0.25]. Compared with the Extension-on-the-beat condition, the SD of interpersonal phase angles in the Flexion-on-the-beat condition was smaller at beat rates from 100 to 160 bpm (*ps* < 0.05). As expected, the interpersonal phase relations were less variable with visual coupling in both the Flexion-on-the-beat condition [*F*(1, 15) = 17.83, *p* < 0.01] and the Extension-on-the-beat condition [*F*(1, 15) = 16.46, *p* < 0.01]. In sum, the analysis of the mean and SD of interpersonal phase relations indicated that individuals synchronised their movements when they could see other.

Control analyses based on permutated data showed that the main effect of permutation [*F*(2, 30) = 23.24, *p* < 0.001, ${\eta }_{p}^{2}$ = 0.61], coordination pattern [*F*(1, 15) = 57.06, *p* < 0.001, ${\eta }_{p}^{2}$ = 0.79], and beat rate [*F*(2.70, 40.50) = 2.96, *p* = 0.04, ${\eta }_{p}^{2}$ = 0.17]. The mean interpersonal phase angles were closer to 0° in the Face-real condition in comparison with the Back-real condition (*p* < 0.001) and the Face-pseudo condition (*p* < 0.001), and not significantly different between the Back-real and Face-pseudo conditions (*p* = 0.56). The SD of interpersonal phase angles was also smaller in the Face-real condition than that in the Back-real condition (*p* < 0.01) and the Face-pseudo condition (*p* < 0.001), and not significantly different between the Back-real and Face-pseudo conditions (*p* = 0.56). There were no significant three-way and two-way interactions including the permutation factor. These results indicated that changes in interpersonal phase relations in the Face-to-Face condition were due to the continuous visual coupling and not to changes in other cognitive processes.

### Auditory-motor coordination

**Figure 3:**
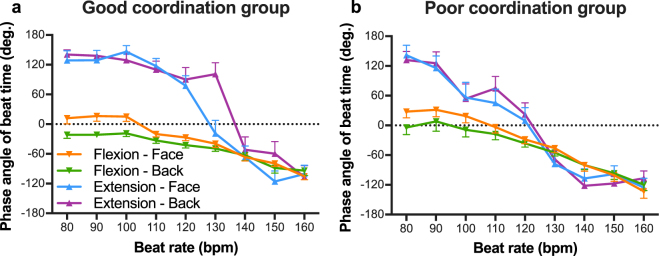
Mean phase angle of beat time in the good coordination group (**a**) and the poor coordination group (**b**) as a function of beat rate. Relative phase values equal to 0° indicate flexion on the beat and values equal to 180° indicate extension on the beat. The range from −180° to 0° is flexion phase and from 0° to 180° is extension phase. Vertical bars represent between-participant standard error. Lower error bars are for the Flexion-on-the-beat condition, and upper error bars are for the Extension-on-the-beat condition.

Consistent with previous work^25^, the phase angle of beat time in the Extension-on-the-beat condition exhibited phase transitions to the Flexion-on-the-beat pattern at beat rates faster than 90 bpm in both the good coordination group (Fig. 3a) and the poor coordination group (Fig. 3b). The ANOVA on these data revealed significant main effects of group [*F*(1, 30) = 6.62, *p* = 0.02, ${\eta }_{p}^{2}$ = 0.18], coordination pattern [*F*(1, 30) = 101.84, *p* < 0.001, ${\eta }_{p}^{2}$ = 0.77], and beat rate [*F*(3.93, 117.89) = 134.86, *p* < 0.001, ${\eta }_{p}^{2}$ = 0.82], and a non-significant main effect of orientation [*F*(1, 30) = 0.00, *p* = 0.97]. The three-way interaction between group, coordination pattern, and beat rate was significant [*F*(4.01, 120.14) = 3.28, *p* = 0.01, ${\eta }_{p}^{2}$ = 0.10]. Two-way interactions between coordination pattern and beat rate were significant in both good coordination group [*F*(8, 240) = 17.17, *p* < 0.001] and poor coordination group [*F*(8, 240) = 12.18, *p* < 0.001]. There were significant differences between coordination patterns from 80 to 130 bpm in good coordination group (*ps* < 0.001) and from 80 and 120 bpm in poor coordination group (*ps* < 0.05), indicating that both groups could perform the two instructed coordination patterns distinguishably at slower beat rates.

**Figure 4:**
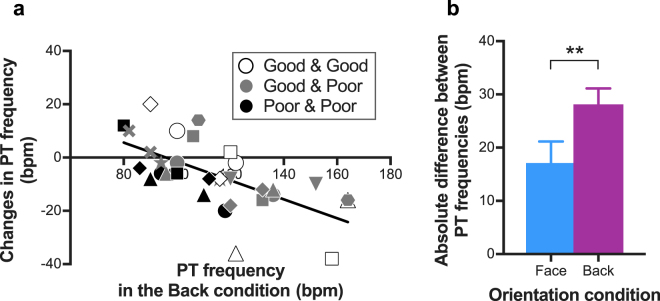
Correlation between phase transition (PT) frequency in the Back-to-back condition and changes with visual coupling in PT frequency (**a**) and absolute difference of PT frequencies within a pair (**b**). In (**a**), each marker represents an individual participant. The shading designates the type of pair on the variability of auditory-motor coordination. Pairs where both participants were designated as good at coordination with the beat are shown in white. Pairs where both participants were designated as poor at coordination are shown in black. Mixed pairs with a good coordination participant and a poor coordination participant are shown in grey. Shapes represent each pair within each pair type. Thus, two markers whose colour and shape are same indicate paired participants. The trend line represents a linear regression line. In (**b**), vertical bars represent between-participant standard error.

To explore the effect of visual coupling on phase transition frequency depending on individual coordination skill, we calculated the Pearson’s correlation coefficient between inherent phase transition frequency (under the Back-to-back condition) and the changes in phase transition frequency with visual coupling (Fig. 4a). A significant negative correlation was found, *r*(30)* = *−0.65, *p <* 0.001, *r* ^*2*^ = 0.42. Therefore, while interacting visually, phase transitions occurred at higher beat rates for participants whose inherent phase transition frequency was relatively low, whereas transitions occurred at lower beat rates for participants whose inherent phase transition frequency was relatively high. In other words, visual coupling was associated with a reduction in the difference between phase transition frequencies within a pair. In fact, as predicted, the absolute difference between phase transition frequencies within a pair was significantly smaller in the Face-to-face condition than in the Back-to-back condition (Fig. 4b), *t*(15) = 3.19, *p* < 0.01, indicating that participants tended to match their movements and individual auditory-motor coordination when they could see each other.

**Figure 5:**
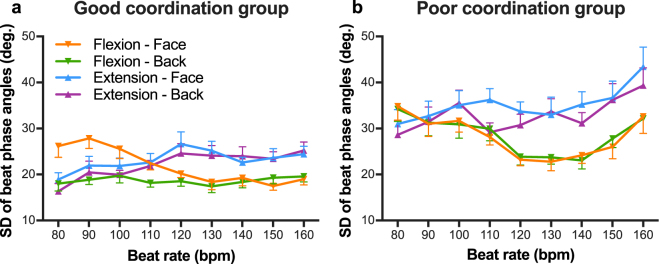
Mean standard deviation of beat phase angles in the good coordination group (**a**) and the poor coordination group (**b**) as a function of beat rate. Vertical bars represent between-participant standard error. Lower error bars are for the Flexion-on-the-beat condition, and upper error bars are for the Extension-on-the-beat condition.

Figure 5 shows the mean SD of beat phase angles as a function of beat rate in the good coordination group (a) and the poor coordination group (b). The ANOVA on these data yielded significant main effects of group [*F*(1, 30) = 60.61, *p* < 0.001, ${\eta }_{p}^{2}$ = 0.67], coordination pattern [*F*(1, 30) = 23.34, *p* < 0.001, ${\eta }_{p}^{2}\,$= 0.44], and orientation [*F*(1, 30) = 7.52, *p* = 0.01, ${\eta }_{p}^{2}$ = 0.20]. There was also a significant three-way interaction between group, orientation, and coordination pattern [*F*(1, 30) = 10.23, *p* < 0.01, ${\eta }_{p}^{2}$ = 0.25]. The two-way interaction between coordination pattern and orientation was significant in both good coordination group [*F*(1, 30) = 4.43, *p* = 0.04] and poor coordination group [*F*(1, 30) = 5.84, *p* = 0.02]. Simple effect tests revealed that, for the poor coordination group, the SD of beat phase angles in the Flexion-on-the-beat condition was smaller than in the Extension-on-the-beat condition in both Face-to-face and Back-to-back conditions [*F*(1, 30) = 27.68, *p* < 0.001 and *F*(1, 30) = 11.66, *p* < 0.01, respectively]. In the good coordination group, the SD of beat phase angles was significantly different between coordination patterns in the Back-to-back condition [*F*(1, 30) = 7.77, *p* < 0.01], but not in the Face-to-face condition. Simple effect tests also revealed that the sight of a partner increased the variability of the Extension-on-the-beat pattern in the poor coordination group [*F*(1, 30) = 6.74, *p* = 0.01]and the variability of the Flexion-on-the-beat pattern in the good coordination group [*F*(1, 30) = 9.86, *p* < 0.01].

**Figure 6:**
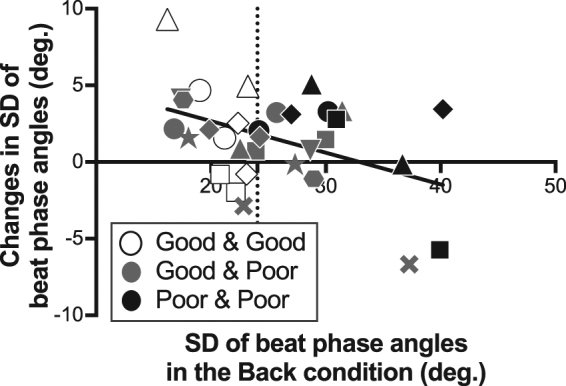
Correlation between SD of beat phase angles in the Back-to-back condition and changes with visual coupling in SD of beat phase angles. Each marker represents an individual participant. The shading designates the type of pair. Pairs where both participants were designated as good at coordinating with the beat are shown in white. Pairs where both participants were designated as poor at coordination are shown in black. Mixed pairs with a good coordination participant and a poor coordination participant are shown in grey. Shapes represent each pair within each pair type. The solid black line represents a linear regression line. The vertical dotted line indicates the median value.

To explore the effect of visual coupling on the variability of auditory-motor coordination depending on individual coordination skill, the Pearson’s correlation coefficient was calculated between the inherent SD of beat phase angles (under the Back-to-back condition) and the changes in SD of beat phase angles due to visual coupling (Fig. 6). The correlation coefficient was significant, *r*(30)* = *−0.43, *p =* 0.01, *r* ^*2*^ = 0.19. Accordingly, the sight of a partner reduced the variability of auditory-motor coordination in the poor coordination group (e.g. black markers in Fig. 6), while it increased variability in the good coordination group (e.g. white markers in Fig. 6).

## Discussion

The current study investigated the interplay between the processes governing unintentional and intentional coordination across different modalities. Previous research reported that interpersonal entrainment occurs spontaneously or unintentionally between visually coupled participants in a range of joint tasks, with either 0° or 180° intermittent phase relations being most common^8–13^. Spontaneous in-phase coordination can occur additionally to a shared visual-pacing signal, producing high levels of interpersonal synchrony^12^. Despite the fact that participant pairs in our study were only instructed to coordinate their movements with the beats (not with each other), results showed interpersonal phase relations became closer to 0° and less variable while performing the auditory-motor coordination task with a partner. Our results indicated that these changes in interpersonal phase relations in the Face-to-Face condition were due to the continuous visual coupling and not to changes in other cognitive processes (e.g., attention load or internal motor representation of the partner), as they were not observed when trials in the Face-to-Face condition were permutated within pairs.

Interpersonal entrainment is induced not only by visual information of a partner^9,13^, but also by partner-related auditory^14,36^ and tactile information^11^. Demos *et al*.^14^ demonstrated that two participants hearing each other’s rocking sounds increased the occurrence of interpersonal entrainment, even in the absence of visual coupling. Paired participants in our experiment, however, performed the task simultaneously in silence to a regular beat, meaning that our results were purely due to visual coupling. Interestingly, interpersonal entrainment preferentially occurred at lower beat rates, which might be due to more visual information about a partner being picked-up within a cycle with slow movement frequencies.

In the current experiment, interpersonal phase relations were not exactly 0° despite the fact that both participants within a pair were required to do the same task with the same beats at the same tempo in the same condition. This may be the case because we asked participants to flex or extend their knees with beats in a comfortable way, leaving room for some degree of idiosyncratic performance. Indeed, there were individual differences in the comfortable coordination pattern in the flexion-on-the-beat and the extension-on-the-beat patterns, which may have made interpersonal phase relations deviate from 0°. Our findings showed that visual coupling decreases these individual differences.

Our experimental setting may have been conducive to strong visual coupling and the occurrence of interpersonal entrainment. In the study of Shockley and colleagues^15^, visual interaction between paired participants solving puzzles via free conversation did not produce interpersonal postural entrainment. Participants in this earlier study did not stand face-to-face, but they stood in front of the board on which pictures for the puzzle game were presented, and a confederate stood next to the board. Visual coupling during interpersonal coordination is known to be generally weak, and peripheral vision makes it even weaker^11,13^. However, foveal vision or standing face-to-face facilitates interpersonal entrainment even under unexpected perturbation^37^, or when participants are explicitly instructed to not coordinate their movements with each other^7^. It is thus possible that the use of central vision favoured the occurrence of interpersonal entrainment in the present experiment. Furthermore, high rhythmicity and large amplitude movements can facilitate spontaneous visuo-motor coordination^16–18^. If the type of movements is complex or less repetition, visual coupling seems not to facilitate interpersonal entrainment even participants were asked to move together with the partner^38^. Accordingly, in our experimental setting, in addition to the use of the central vision, it is possible that the type of movements investigated –rhythmic movements with large amplitude –facilitated the occurrence of interpersonal entrainment.

Several studies have reported that social factors, such as individual personality characteristics and likeability for a partner, influence upon the extent to which interpersonal phase relations become closer to 0° during visual interaction^3,39–43^. These social factors can also affect individual dynamics of auditory-motor coordination asymmetrically. In the context of dancing and music education, for example, a teacher might strongly influence a learner, while the learner has less effect on the teacher. Again, such asymmetrical effects of visual coupling could be different based on interpersonal relations, individual personality characteristics, and mutual likeability. In the current study, we focused on the general effect of interpersonal entrainment on individual auditory-motor coordination. Although we did not find any significant interaction between visibility of the partner and pair characteristics, such as partner-familiarity and gender-match (see Supplementary Table S1–S3), this could be due to reduced sample sizes. Future work needs to address specific effects of social factors on individual auditory-motor coordination skill and interpersonal entrainment.

To test the hypothesis that the effect of visual coupling differs depending on individual coordination skill, we divided our participant sample into a good coordination group and a poor coordination group based on the variability of auditory-motor coordination in the Back-to-back condition. We used this classification based on the assumption that participants in the Back-to-back condition performed auditory-motor coordination with lowest degree of interference, and that this performance could therefore be considered as a measure of each individual’s inherent performance level.

In line with previous research^25^, phase transitions from the Extension-on-the-beat pattern to the Flexion-on-the-beat pattern were observed as beat rate increased regardless of whether or not participants were visually coupled in both coordination skill groups. Coordination dynamics, including phase transition frequencies, vary between participants due to different biomechanical constraints and training^25,28,44^. We found that phase transitions occurred earlier or later when participants were visually coupled than without visual coupling, leading to greater in-phase coordination in the former case. In related work, Varlet *et al*.^12^ demonstrated that visual coupling increases the variability of individual visuo-motor coordination, although vision of the target may have conflicted with vision of the partner in that particular study. Our results also indicated that visual coupling increased the variability of the Flexion-on-the-beat pattern in the good coordination group and the variability of the Extension-on-the-beat pattern in the poor coordination group. However, as shown in Fig. 6, the sight of a partner helped the poor coordination group to better coordination, as indicated by overall reduced variability of auditory-motor coordination. These results demonstrate that, rather than increasing coordination variability, visual coupling compensates for the difference in coordination variability between paired participants. This supports the hypothesis that spontaneous interpersonal coupling makes individuals susceptible to a partner’s movement variability, especially for good coordination group (who may have relatively high perceptual sensitivity for rhythmic stimuli). Taken together, our results show that visual coupling bridges the difference in inherent dynamics of auditory-motor coordination between two individuals.

Previous studies on factors that affect the dynamics of auditory-motor coordination have reported the influence of neuromuscular-skeletal properties^45^, salient perceptual information^46^ and environmental constraints such as gravity^47^. Studies on bimanual coordination demonstrated that coupling with environmental rhythms modulates intra-personal coordination dynamics^48–51^, including coupling with rhythms produced by other people^52^. The effect of interpersonal entrainment on individual sensorimotor coordination has been reported to date only in visuo-motor coordination^12^. The current study extends these previous findings by showing that interpersonal entrainment induced via visual coupling can also modulate the dynamics of auditory-motor coordination.

Several studies have found that sensorimotor coordination is less variable with auditory rhythms than with visual rhythms (consisting of flashing lights) presented together with distractor sequences in the other sensory modality^53^. There is, however, no difference in the effect of distractor modality when visual rhythms are presented with continuous trajectories (such as a bouncing ball)^54^. Although the instructions were different in these previous studies, where participants were asked to maintain a specific coordination pattern, from the current study, where participants were asked to produce the most comfortable pattern, our participants might have experienced similar conflict between modalities. Furthermore, previous studies have reported that biological movement stimuli have stronger effects on human motor control than non-biological stimuli^55,56^. In the present study, the visual rhythm was continuous and consisted of a human partner, raising the possibility that our participants experienced stronger effects of visual stimuli than previous studies using non-biological movement stimuli.

The main novelty of the current study lies in the observed impact of visual coupling, which induced interpersonal entrainment upon individual actor-environment coordination in terms of coordination stability. Previous research has not addressed the impact upon individual action-perception coordination skill—which is a critical ability for music, dancing, sports and daily life—although a growing number of studies has reported effects of interpersonal entrainment on psychological indices such as affiliation, likeability, social bonding, self-esteem and social behaviour^39,57–59^. Our findings thus extend the current literature regarding the effect of interpersonal entrainment on agent-environment coordination skill. Specifically, interpersonal visual coupling reduces individual differences in phase transition frequency and the variability of auditory-motor coordination.

In summary, the present study yielded two novel findings. Visual coupling encourages interpersonal entrainment during the performance of auditory-motor coordination tasks and reduces individual differences in the dynamics of auditory-motor coordination. This is consistent with the more general view that complex motor behaviours spontaneously emerge and change according to reciprocal interactions between individuals and their environment^60^. Thus, even if we intend to reproduce the same action, our performance might be unintentionally modulated when we are in a new environment interacting with new co-actors. In group dance in social settings, for example, seeing each other may enable individuals to achieve unity and harmony of group performance by reducing differences in individual performances. There may, however, be asymmetries in the way in which this is achieved, with visual interaction helping individuals who are poor at auditory-motor coordination but degrading the performance of individuals who are good at auditory-motor coordination.

## Electronic supplementary material

Supplementary Info
