# Supplementary material for: Modulation of individual auditory-motor coordination dynamics through interpersonal visual coupling
Source: Sci Rep. 2017 Nov 24;7:16220. doi: 10.1038/s41598-017-16151-5 (PMC5701212; doi:10.1038/s41598-017-16151-5)
Supplement: Supplementary file 1 — Supplementary Info [file 41598_2017_16151_MOESM1_ESM.doc]

**Supplementary Information**

**Title:** Modulation of individual auditory-motor coordination dynamics
through interpersonal visual coupling

**Author list:** Kohei Miyata^*^, Manuel Varlet, Akito Miura, Kazutoshi Kudo
and Peter E. Keller

**The effects of gender-match and partner-familiarity on the present data**

Previous studies suggest that the pair characteristics, such as partner-familiarity and gender-match can affect interpersonal entrainment. Participant pairs in the current study consisted of 7 mixed pairs and 9 female pairs. Members of two female pairs knew each other from before the experiment, so they were classified as having partner-familiarity. In order to explore the effect of gender-match and partner-familiarity, we performed separate 5-way ANOVAs with 2 between-subject factors – gender-match and partner-familiarity – and three within-subject factors – coordination pattern, orientation condition, and beat rate – on (1) the mean relative phase, (2) the mean SD of relative phase between the two participants, (3) the mean phase angle of beat time, and (4) the mean SD of beat phase angles. A 3-way ANOVA with two between-subject factors – gender-match and partner-familiarity – and one within-subject factor was performed on the absolute difference between transition frequencies within a pair.

There were no significant main effects of gender-match and partner-familiarity for any dependent measures. An interaction between gender-match and coordination was significant for mean interpersonal relative phase [*F*(1, 13) = 16.27, *p* = .001,
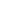
= .56]. Simple effect tests revealed that the mean interpersonal relative phase of mixed pairs was closer to 0° than that of female pairs in the Extension-on-the-beat condition [*F*(1, 13) = 4.78, *p* = .048,
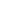
= .27]. Furthermore, a significant interaction between gender-match and beat rate was revealed for the SD of beat phase angles [*F*(3.17, 91.83) = 3.00, *p* = .032,
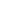
= .06]. Simple effect tests showed that the SD of beat phase angles was smaller in female pairs than in mixed pairs [*F*(1, 29) = 4.81, *p* = .036,
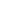
= .14]. There was no significant interaction between familiarity and other factors.

These results showed that the effect of gender-match in mean interpersonal phase relations and the SD of beat phase angles occurred without interaction with orientation. Therefore, our results indicated that visual coupling affects interpersonal phase relations and individual dynamics of auditory-motor coordination irrespective of pair characteristics, such as partner-familiarity and gender-match. Caution in the interpretation of the results remains necessary, however, as these analyses are based on reduced sample sizes.


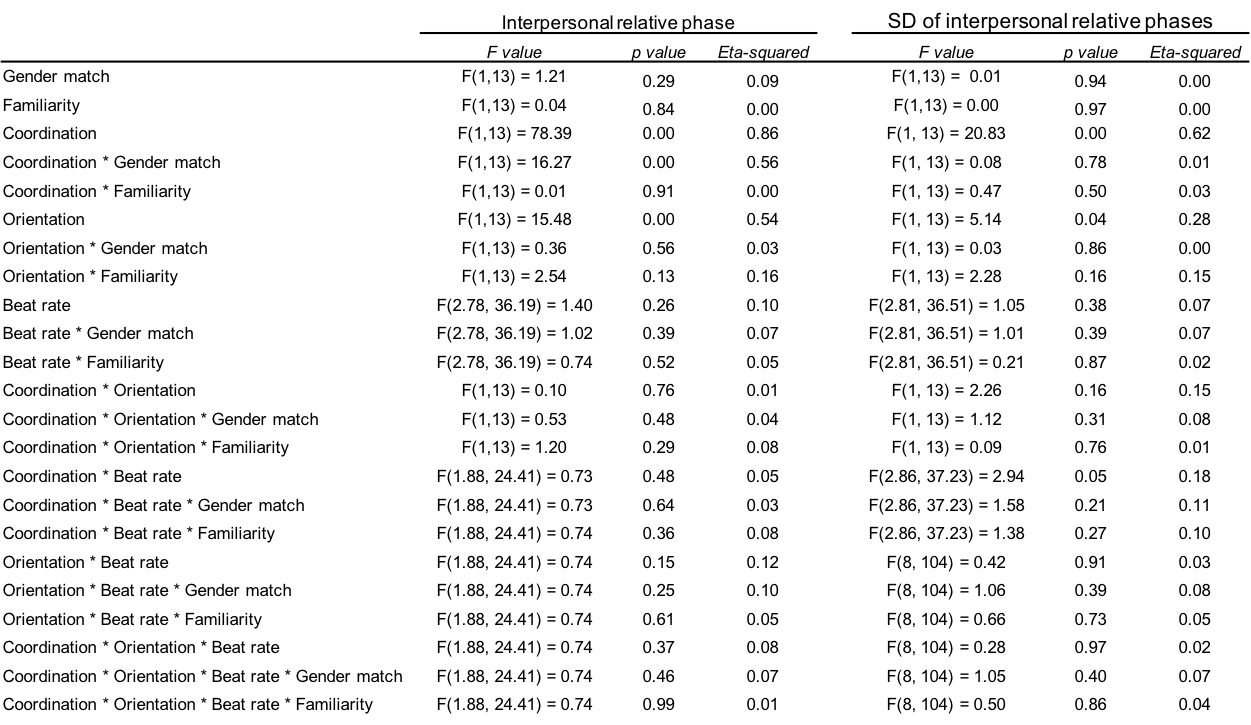


*Supplementary Table S1.* The mean and SD of interpersonal phase relations


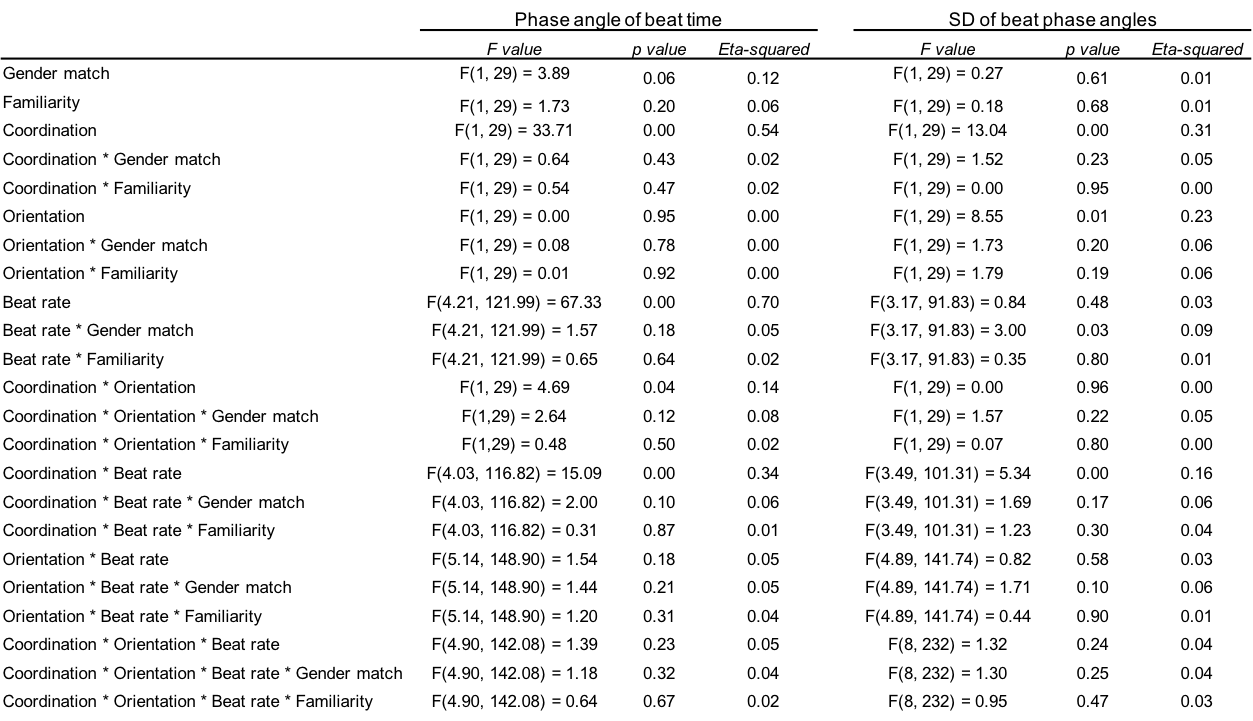


*Supplementary Table S2.* The mean and SD of beat phase angles


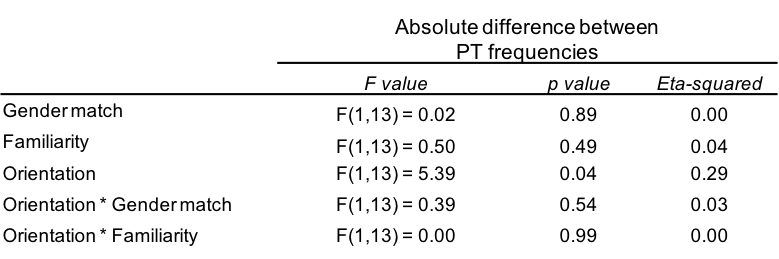


*Supplementary Table S3*. Absolute difference between PT frequencies
